# Therapeutic Prospects of Cannabidiol for Alcohol Use Disorder and Alcohol-Related Damages on the Liver and the Brain

**DOI:** 10.3389/fphar.2019.00627

**Published:** 2019-05-31

**Authors:** Julia De Ternay, Mickaël Naassila, Mikail Nourredine, Alexandre Louvet, François Bailly, Guillaume Sescousse, Pierre Maurage, Olivier Cottencin, Patrizia Maria Carrieri, Benjamin Rolland

**Affiliations:** ^1^Service Universitaire d’Addictologie de Lyon (SUAL), Bron, France; ^2^Université de Picardie Jules Verne, Centre Universitaire de Recherche en Santé, INSERM UMR 1247, Groupe de Recherche sur l’Alcool & les Pharmacodépendances, Amiens, France; ^3^Service des maladies de l’appareil digestif, CHU Lille, Universitéde Lille and INSERM U995, Lille, France; ^4^Service d’Addictologie et d’Hépatologie, GHN, HCL, Lyon, France; ^5^Université de Lyon, UCBL, Centre de Recherche en Neurosciences de Lyon (CRNL), Inserm U1028, CNRS UMR5292, PSYR2, Bron, France; ^6^Laboratory for Experimental Psychopathology (LEP), Psychological Science Research Institute, Université catholique de Louvain, Louvain-la-Neuve, Belgium; ^7^CHU de Lille, Université Lille, service d’addictologie, CNRS, UMR 9193, SCALab, équipe psyCHIC, Lille, France; ^8^INSERM, UMR_S 912, Sciences Economiques & Sociales de la Santé et Traitement de l’Information Médicale (SESSTIM), Marseille, France

**Keywords:** alcohol use disorder, alcohol-related damage, cannabidiol, liver fibrosis and cirrhosis, neuroprotection, addiction

## Abstract

**Background:** Cannabidiol (CBD) is a natural component of cannabis that possesses a widespread and complex immunomodulatory, antioxidant, anxiolytic, and antiepileptic properties. Much experimental data suggest that CBD could be used for various purposes in alcohol use disorder (AUD) and alcohol-related damage on the brain and the liver.

**Aim:** To provide a rationale for using CBD to treat human subjects with AUD, based on the findings of experimental studies.

**Methods:** Narrative review of studies pertaining to the assessment of CBD efficiency on drinking reduction, or on the improvement of any aspect of alcohol-related toxicity in AUD.

**Results:** Experimental studies find that CBD reduces the overall level of alcohol drinking in animal models of AUD by reducing ethanol intake, motivation for ethanol, relapse, anxiety, and impulsivity. Moreover, CBD reduces alcohol-related steatosis and fibrosis in the liver by reducing lipid accumulation, stimulating autophagy, modulating inflammation, reducing oxidative stress, and by inducing death of activated hepatic stellate cells. Finally, CBD reduces alcohol-related brain damage, preventing neuronal loss by its antioxidant and immunomodulatory properties.

**Conclusions:** CBD could directly reduce alcohol drinking in subjects with AUD. Any other applications warrant human trials in this population. By reducing alcohol-related steatosis processes in the liver, and alcohol-related brain damage, CBD could improve both hepatic and neurocognitive outcomes in subjects with AUD, regardless of the individual’s drinking trajectory. This might pave the way for testing new harm reduction approaches in AUD, in order to protect the organs of subjects with an ongoing AUD.

## Introduction

Alcohol use disorder (AUD) is an addictive disorder characterized by a progressive loss of control upon alcohol use. AUD consists of several clinical criteria that include alcohol tolerance, withdrawal symptoms, craving, as well as medical and psychosocial consequences. AUD is responsible for a severe burden of disease. Worldwide, AUD causes more than 3 million deaths every year, which represents 5% of all deaths (World Health Organization, 2018). More specifically, subjects with AUD may be affected by the consequences of recurrent alcohol abuse on the body, including alcohol-related liver disease (ARLD), and alcohol-related brain injury (ARBI).

ARLD is a progressive alcohol-induced liver injury, which starts with an increase in the amount of fat in the liver—a process called steatosis—and continues into a progressive cell loss, fibrosis, and hepatic insufficiency—a process called cirrhosis (O’Shea et al., 2010). ARLD may result in severe liver failure, and represents a major risk factor for liver cancer. Overall, alcohol-attributable liver damage is responsible for 493,300 deaths every year, and 14,544,000 disability adjusted life years (DALYs), representing 0.9% of all global deaths and 0.6% of all global DALYs all over the world (Rehm et al., 2013). In subjects with ARLD, preventing the transition from steatosis to cirrhosis is a major treatment goal, and this usually requires to stop or to dramatically reduce the average amount of consumed alcohol in the long term (European Association for the Study of the Liver A et al., 2018). AUD also affects the brain, through ARBI. Subjects with AUD display reduced gray matter volumes and reduced cortical thickness, as well as increased ventricular volumes, when compared to matching healthy controls (Bühler and Mann, 2011). The most significant reductions in grey matter volumes are observed in the corticostriatal–limbic circuits, including the insula, superior temporal gyrus, dorso-lateral prefrontal cortex, anterior cingulate cortex, striatum, and thalamus (Bühler and Mann, 2011). Cognitive functions associated with these brain areas (e.g., executive functions, working memory, emotion recognition, or long-term memory) are impaired in subjects with AUD (Stavro et al., 2013). Generally, cognitive dysfunctions start to improve quickly after alcohol withdrawal, but patients substantially recover only within the first weeks to months of alcohol abstinence, and sometimes remain impaired (Stavro et al., 2013; Schulte et al., 2014). Similarly, the recovery of structural brain alterations can be highly variable depending on brain areas and individual features (Durazzo et al., 2015; Zou et al., 2018). Overall, both ARLD and ARBI involve alcohol-related inflammatory processes (Mandrekar and Ambade, 2014; Neupane, 2016). Current medications for reducing alcohol drinking or supporting alcohol abstinence in AUD subjects are still insufficiently effective at a population level, and new therapeutic prospects are needed (Rolland et al., 2016; Soyka and Müller, 2017). Moreover, no drug for reducing alcohol-related harms, either on the brain or the liver, has ever been studied.

Cannabidiol (CBD) is a natural constituent of *Cannabis sativa*. Unlike tetra-hydrocannabinol (THC), CBD has no psychotomimetic properties. However, CBD exerts several important effects on the central nervous system, including anxiolytic, antipsychotic (Iseger and Bossong, 2015), analgesic, or antiepileptic effects (Campos et al., 2016; Lee et al., 2017). In this respect, an oromucosal spray with CBD and THC in a 1:1 ratio (SATIVEX^®^, GW Pharmaceuticals) has been approved in Canada as a treatment for multiple sclerosis spasticity (Keating, 2017) since 2005, and is now approved in 22 countries worldwide.

More recently, CBD has been approved in the US for seizures prevention in Dravet and Lennox–Gastaut syndromes, and will therefore be available for clinical practice very soon (Food and Drugs Administration, 2018). Due to its action on cognitive processes and anxiety regulation, CBD is also increasingly considered as a potential treatment for other neuropsychiatric disorders, including anxiety, depression, and substance use disorders (Campos et al., 2016; Lee et al., 2017). In addition to its actions on the brain, CBD has systemic effects, through its complex immunomodulatory and antioxidant properties (Booz, 2011). This has raised increasing interest in CBD for various inflammatory or immunological diseases, such as cancer (Massi et al., 2013), neurodegenerative diseases (Fernández-Ruiz et al., 2013; Karl et al., 2017), colitis (Jamontt et al., 2010), cardiovascular diseases (Stanley et al., 2013), and diabetes (Gruden et al., 2016).

CBD is a weak, noncompetitive, negative allosteric modulator of cannabinoid-1 (CB1) receptors (Pertwee, 2008; Laprairie et al., 2015; Tham et al., 2019), however, a large part of the pharmacological action of CBD seems to be based on mechanisms that do not involve cannabinoid receptors. For example, the molecular mechanisms through which CBD prevents seizures are currently debated on, but several potential molecular targets other than cannabinoid receptors have been identified. In particular, CBD is a partial antagonist of G protein-coupled receptor 55 (GRP55), identified as an endocannabinoid target (Ryberg et al., 2009), which could be involved in the decrease of neuronal excitability, through an action on gamma-aminobutyric acid-ergic (GABAergic) neurotransmission (Devinsky et al., 2014; Musella et al., 2017; Chen et al., 2018). CBD also regulates calcium (Ca2+) homeostasis by acting on mitochondria stores (Ryan et al., 2009), and blocks low-voltage-activated (T-type) Ca2+ channels, modulating intracellular calcium levels (Ross et al., 2008). Other hypotheses include inhibition of anandamide hydrolysis *via* fatty acid amide hydrolase (FAAH) (Watanabe et al., 1998; Massi et al., 2008; Leweke et al., 2012), activation of peroxisome proliferator-activated receptor γ (PPAR-γ) (Devinsky et al., 2014), positive allosteric modulation of serotonin 1A receptors (5-HT1A receptors) (Rock et al., 2012), activation of transient receptor potential vanilloid type 1 (TRPV1), and reduction of adenosine reuptake increasing adenosine levels (Carrier et al., 2006; Zhornitsky and Potvin, 2012).

The systemic immunomodulatory and antioxidant properties of CBD appear to be based on complex mechanisms. CBD acts on many cellular pathways of inflammation, such as the nuclear factor kappa-light-chain-enhancer of activated B cells (NF-κB) pathway (Rajesh et al., 2010; Juknat et al., 2012; Khaksar and Bigdeli, 2017), as well as the interferonβ/signal transducer and activator of transcription proteins (IFNβ/STAT) pathway (Juknat et al., 2012). Through activation of adenosine receptor A2a, and inhibition of adenosine reuptake (Carrier et al., 2006; Castillo et al., 2010), CBD can modulate the activity of multiple inflammatory cells, including neutrophils, macrophages, or T-cells. CBD also decreases the production of inflammatory mediators such as interferon-c (IFN-c), interferon-γ (IFN-γ) (Lee and Erdelyi, 2016), tumor necrosis factor α (TNF-α) (Magen et al., 2009; Rajesh et al., 2010; Khaksar and Bigdeli, 2017; Wang et al., 2017), interleukin (IL)-1β (IL-1β) (Pazos et al., 2013; Wang et al., 2017), IL-6 (Lee and Erdelyi, 2016), and the expression of intercellular adhesion molecule 1 (ICAM1) and vascular cell adhesion molecule 1 (VCAM1) (Rajesh et al., 2010). Furthermore, CBD decreases caspase 9 (Castillo et al., 2010) and caspase 3 activation (Iuvone et al., 2004; Rajesh et al., 2010; Da Silva et al., 2014; Santos et al., 2015), which are factors involved in apoptosis. CBD up-stimulates anti-inflammatory cytokines IL-10 (Kozela et al., 2017). Finally, CBD activates the PPAR-γ, a nuclear receptor that plays a central role in the regulation of metabolic and inflammatory cell processes, including those leading to apoptosis (O’Sullivan and Kendall, 2010).

Because of its various effects on the brain and on systemic inflammation, CBD involves a large potential array of complementary therapeutic applications in AUD. First, CBD could help patients with AUD reduce their level of alcohol drinking. Second, by modulating the inflammatory processes in the liver, CBD could reduce alcohol-induced liver steatosis and fibrosis, thus constituting a novel harm reduction agent among subjects with AUD, particularly among those who still exhibit heavy drinking. Third, CBD could reduce ARBI. The aim of this narrative review is to offer a comprehensive overview of the current body of evidence about these three specific applications of CBD in subjects with AUD or animal models of AUD, and to discuss what should be the next steps of research on these topics.

## Methods

A narrative review was performed after a systematic search on PubMed, using the following algorithm: “cannabidiol AND (alcohol OR ethanol).”

On the basis of the 143 studies published between 1974 and June 2018, 26 original studies were included in the present review. Additional articles useful for the rationale of the review were selected from the reference list of initially selected studies, or using independent search results on PubMed.

Results are sorted in three independent sections: cannabidiol for reducing alcohol drinking, cannabidiol for reducing alcohol-related liver inflammation, and cannabidiol for reducing alcohol-related brain injuries.

## Cannabidiol for Reducing Alcohol Drinking Levels

CBD effects on alcohol drinking were tested in preclinical studies using several procedures to investigate AUD, including propensity to drink ethanol with the two-bottle choice or the operant self-administration procedure, and behavioral sensitization. Four main studies have been published so far, and they provide thorough and congruent evidence that, in rodents, CBD can reduce ethanol intake, motivation for ethanol, relapse, reinstatement after extinction, as well as the levels of anxiety and impulsivity correlated with ethanol intake.

A first study in male C57BL/6J mice, an ethanol-preferring strain, demonstrated that the administration of CBD reduced reinforcing properties, motivation, and ethanol relapse (Viudez-Martínez et al., 2018). Increasing doses of CDB (30, 60, and 120 mg/kg) administered intraperitoneally (i.p.) progressively decreased both ethanol preference (from 75% to 55%) and intake (from about 6 g of pure ethanol/kg body weight/day to 3.5 g/kg/day) in a two-bottle choice paradigm (water versus 8% ethanol solution). The results were confirmed in an operant paradigm in which mice had to press a lever to get access to 36 mL of 8% ethanol solution. In the operant paradigm, animals had to work (press a lever) to get access to ethanol; this is useful to assess motivation to drink ethanol, because the price to pay (effort) can be increased by the experimenter. In the context of this operant paradigm that includes a saccharin fading phase, administration of the CBD-controlled release microparticle subcutaneous (s.c.) formulation (30 mg/kg/day, s.c.) significantly reduced the number of active lever presses by about 40% in a fixed-ratio one schedule (one press required to get ethanol) as well as in a more demanding fixed-ratio three schedule (three presses required to get ethanol). It also reduced motivation to drink ethanol by about 50% in a progressive ratio schedule, and relapse by about 30% after an extinction session with a 120 mg/kg i.p. dose. It had no effect on water reinforcement or motivation. In addition, CBD reduced 3.0 g/kg ethanol-induced hypothermia and 4.0 g/kg ethanol-handling-induced convulsions but did not have any effect on blood ethanol concentration. CBD treatment was associated with changes in gene expression of key targets closely related to AUD. A single administration of CBD (30 mg/kg/day, s.c.) during oral ethanol self-administration decreased gene expression of Oprm1, GPR55, and CB1 receptor in the nucleus accumbens (NAc), while CB2 receptor expression was increased; it also decreased gene expression of gene encoding tyrosine hydroxylase (TH) in the ventral tegmental area (VTA). In a second study, the same authors tested the effect of CBD (20 mg/kg s.c.), of naltrexone (0.7 mg/kg, oral), and of their combination in male C57BL/6J mice using the same operant paradigm (Viudez-Martínez et al., 2018). They found that combining CBD and naltrexone reduces ethanol consumption and motivation to drink ethanol more efficiently than either drug administered alone. 5-HT1A receptor gene expression was reduced in the dorsal raphe nucleus after CBD treatment.

A third study was carried out in male Wistar rats using an operant paradigm in which animals pressed a lever to get a 10% ethanol solution during 30-min sessions (Gonzalez-Cuevas et al., 2018). CBD was administered transdermally (gel concentration: 2.5 g CBD/100 g gel) to avoid low oral bioavailability (∼6%) and conversion into psychoactive cannabinoids in gastric fluid. Transdermal CBD produces stable and sustained plasma CBD levels. Rats were trained for 2 weeks during the sweet solution fading phase, then trained for only 10 days under a fixed ratio 1 schedule and finally, extinction sessions were carried out (i.e., sessions without ethanol and ethanol-associated cues). After extinction and baseline (vehicle treatment) reinstatement, the effect of 15 mg/kg CBD (delivered every 24 h over a 7-day treatment phase) was tested on reinstatement induced either by context, by pharmacological stress (yohimbine 1.25 mg/kg i.p.), or by physical stress (footshock). CBD reduced the number of responses during context-induced reinstatement (∼50% decrease) on sessions (days) 1, 4, and 7 of the treatment phase. CBD effect was long lasting, since the 50% reduction was still visible 3, 18, 48, and even 138 days (sessions) after the CBD treatment phase. CBD treatment was also efficient on stress-induced reinstatement and particularly on the one induced by yohimbine pharmacological stress. As for the effect of the context-induced reinstatement, the stress-induced reinstatement was strongly reduced 138 days after CBD treatment. Since the benefit of CBD treatment may come from its anxiety prevention properties, the authors also tested its effect in the elevated plus maze on rats that had consumed ethanol and ethanol-naïve rats. CBD (15 mg/kg) decreased anxiety in both groups. CBD effects do seem AUD specific since it had no effect on reward seeking motivated by palatable sweet solution. Moreover, AUD is associated with impulsivity in humans and impaired impulse control is a risk factor for relapse. Interestingly, the authors tested the effect of CBD (15 mg/kg) on impulsivity in rats with a history of ethanol intake using a delay discounting task (preference for delayed large over small immediate reward as a function of delay time). Preference for delayed large reward was significantly lower in rats with ethanol history compared to ethanol-naïve rats and this effect was fully reversed by CBD.

A fourth study in male mice tested the effect of CBD on behavioral sensitization to the motor stimulant effects of ethanol (Filev et al., 2017). Behavioral sensitization is a relevant animal model used to study the incentive salience sensitization theory of drug addiction. The sensitization to the motor stimulant effects of ethanol may reflect the sensitization to the motivation to consume ethanol during the development of addiction, and may be of particular importance during escalation of drug use and during relapse, since it is a very long-lasting phenomenon (even after a long period of abstinence). Sensitization is considered to be a first step in neuroplasticity associated with drug dependence and may mimic the transition from use to abuse and dependence. In the sensitization model, CBD (2.5 mg/kg) had no effect on the acquisition and expression phases.

In summary, preclinical evidence show that CBD may be of strong therapeutic interest in AUD and could have a significant action on drinking levels in human subjects with AUD, since it is effective on different aspects of the disease (intake, motivation, relapse, anxiety, and impulsivity). However, it should be noted that there are no available data on CBD efficacy in more relevant animal models of AUD, such as binge drinking models (Jeanblanc et al., 2018; Jeanblanc et al., 2019) or in models that use more chronic exposure to ethanol and behaviors linked to addiction (loss of control over intake, compulsive use of ethanol, increased motivation) (Meinhardt and Sommer, 2015). Thus, whether CBD is effective in animal models such as the postdependent state, in which rats drink ethanol for months and are exposed to ethanol vapors in order to induce dependence, is unknown.

## Cannabidiol for Reducing Alcohol-Related Liver Inflammation

Animal studies also demonstrated that CBD could significantly reduce liver steatosis and fibrosis that are induced by both chronic and binge ethanol administrations, based on its antioxidant, immunomodulatory, and lipid metabolic regulation properties.

In ethanol-fed rats and mice hepatic cells (Lim et al., 2011), CBD triggered the activation of an endoplasmic reticulum stress response, leading to the selective death of activated hepatic stellate cells (HSC) through activation of the inositol-requiring enzyme 1/apoptosis signal-regulating kinase 1/c-Jun N-terminal kinase (IRE1/ASK1/JNK) pathway. By contrast, CBD had no effect on HSC in control rats. HSC are involved in the development and progression of liver cirrhosis. As the activation of HSC increases, there is an excessive production of type I collagen, leading to a progressive hepatic fibrosis. The activation mechanism of this pathway was independent from cannabinoid receptors, suggesting that the action of CBD on alcohol-induced liver steatosis is not mediated by this specific pharmacological pathway.

In another study, CBD was demonstrated to reduce binge-alcohol-induced liver damage (Yang et al., 2014). Mice were force-fed with ethanol (30% v/v in saline, 4 g/kg) every 12 h for 5 days. They were then divided into two groups, and injected i.p. 30 min before each ethanol gavage with either CBD (5 mg/kg) or vehicle (Tween 80 2% saline). Eventually, mice were sacrificed, and their serum and liver were collected. CBD prevented the increase in serum aspartate aminotransferase (AST), a marker of liver injury, and significantly attenuated the increase in hepatic triglycerides (TG) level. CBD also stimulated *in vitro* and *in vivo* autophagy, which alleviated lipid accumulation. Finally, CBD decreased ethanol-induced oxidative stress in the liver, and prevented c-Jun N-terminal kinases (JNK) pathway activation, by blocking the increase in JNK phosphorylation. Interestingly, administration of CBD had no effect on control cells injected with vehicle, suggesting a selective mechanism of regulation. Similarly, CBD did not alter the activation of cytochrome P450 E21(CYP2E1), which is supposed to promote steatosis induction. This raises the hypothesis that CBD does not act through this pharmacological pathway.

In an animal model of chronic ethanol feeding and binge ethanol feeding (Wang et al., 2017), mice were fed with a control Lieber–DeCarli diet for 5 days to acclimate them to a liquid diet. Subsequently, a control group was fed with an isocaloric control diet while the other group was fed with a Lieber–DeCarli diet containing 5% ethanol for 10 days, to mimic a chronic ethanol intoxication. On day 11, ethanol and pair-fed mice were respectively force fed with a single dose of ethanol (5 g/kg) or with isocaloric dextrin–maltose. During the 11 days of ethanol exposure, ethanol-fed mice were injected with CBD (5 or 10 mg/kg) dissolved in a vehicle solution (one drop of Tween 80 in 3 mL 2.5% dimethyl sulfide in saline) while control mice were injected with a vehicle solution. Both solutions were administered i.p. CBD reduced hepatic lipids and TG accumulation, neutrophil infiltration, and neutrophil-mediated oxidative injury and inflammation, and attenuated the increase in serum ALT and serum aspartate aminotransferase (AST) levels in ethanol-fed mice. In this group, CBD modulated the ethanol-induced dysregulation of numerous genes and proteins involved in metabolism and liver steatosis, such as key genes of fatty acid biosynthetic and oxidation pathway, mitochondrial pathway, and transcription factor PPAR-α. Furthermore, in the ethanol-fed mice group, CBD attenuated hepatic neutrophils infiltration, oxidative and nitrative stress, decreased several markers of liver inflammation such as TNF-α, the expression of adhesion molecule E-selectin, proinflammatory chemokine and cytokines, and thus, attenuated liver injury induced by chronic plus binge ethanol exposure. None of these effects were found in the pair-fed mice.

Consequently, in both previous studies, CBD reduced ethanol-induced TG accumulation in the liver. The metabolic regulation properties of CBD were also demonstrated in a hepatosteatosis model (Silvestri et al., 2015), both *in vitro* and *in vivo*. Human hepatocyte line 5 cells (HHL-5 cells) were exposed to oleic acid for various periods of time, and coincubated at different times with tetrahydrocannabivarin (THCV) or CBD. CBD and THCV directly reduced accumulated lipids and adipocytes levels *in vitro*. These results were subsequently demonstrated *in vivo*, as CBD (3 mg/kg) was administered for 4 weeks to mice, significantly reducing liver TG content. Neither CB1 nor TRPV1 knockdown inhibited CBD activity, suggesting a mechanism independent from these receptors.

In summary, CBD seems to have valuable therapeutic properties for ethanol-induced liver damage, through multiple mechanisms such as reduction of oxidative stress, modulation of inflammation, death of activated HSC responsible for fibrosis, stimulation of autophagy, and reduction of lipid accumulation responsible for steatosis. These first results accumulating in animal models call for further research in humans.

## Cannabidiol for Reducing Alcohol-Related Brain Damage

Binge and chronic heavy alcohol use are responsible for neuronal damage in specific brain areas, such as the frontal lobe, part of the limbic system, and cerebellum (Bühler and Mann, 2011). Moreover, alcohol induces multiple cognitive deficits, including memory and executive dysfunction (Stavro et al., 2013). Neuroprotective, immunomodulatory, and antioxidant properties of CBD could thus prevent or alleviate some alcohol-related brain damage.

CBD was demonstrated to act as a neuroprotective antioxidant in a binge-ethanol rats model (Hamelink, 2008), in which rats were fed with an alcohol-free diet for 3 days. On day 4, they were administered an ethanol diet (10 to 12% ethanol, 9–12 g/kg/day) every 8 h for 4 days. At the same time, rats received in a double-blind manner either CBD (20 or 40 mg/kg) twice a day, or other tested neuroprotective substances such as antioxidants (butylated hydroxytoluene, α-tocopherol), N-Methyl-D-Aspartate (NMDA) receptor antagonists (dizocilpine, nimodipine, memantine), or diuretics (furosemide, bumetanide, L-644,711). Animals were then sacrificed and the number of degenerating brain cells was determined for each brain tissue section. At the end of the experiment, binge-ethanol rats had lost a significant number of neurons in the hippocampus and in the entorhinal cortex. CBD, at dose range 40 mg/kg coadministered with ethanol, significantly reduced ethanol-induced cell death for both hippocampal granular cells and entorhinal cortical pyramidal cells. Furthermore, CBD was demonstrated to have an antioxidant effect comparable to butylated hydroxytoluene and tocopherol, which significantly decreased ethanol-induced neuronal death in the experiment.

In another study, CBD was delivered transdermally to rats as a treatment for ethanol-induced neurodegeneration (Daniel Liput, 2008). Rats were either administered ethanol (25% w/v) or an isocaloric diet every 8 h for 4 days by intragastric gavage. Plasma levels of ethanol and CBD were measured on day 3. CBD plasma concentration was also measured in trunk blood collected after euthanasia. Fluoro-JadeB (FJB) was used to assess neurodegeneration on brains extracted after euthanasia.

In a first experiment, rats received CBD by daily gel application with different concentrations of CBD (1.0%, 2.5%, 5%) or vehicle, after the third dose of ethanol. Neurodegeneration was visible by FJB+ staining in the entorhinal cortex after 4 days of binge-ethanol intoxication. The 5% CBD gel-treated group showed a 48.8% reduction in the number of FJB+ cells, what trended to statistical significance. In a second experiment, the same model of ethanol intoxication was used. Each group received either ethanol only, vehicle i.p, CBD i.p., or CBD transdermal delivery. CBD administered i.p and transdermally significantly reduced FJB+ cells in the entorhinal cortex compared to the ethanol-only group. However, this effect did not reach statistical significance when compared with the vehicle group.

CBD was also studied in a model of chronic liver disease leading to hepatic encephalopathy (Magen et al., 2009). Bile duct ligation (BDL) was conducted on mice, to mimic biliary liver disease causing elevation of liver enzymes and liver fibrosis, responsible for cognitive and motor impairments. CBD (5 mg/kg) was injected i.p. every day for 4 weeks, starting after surgery. An antagonist of A2a adenosine receptors (A2aR), ZM241385, was injected i.p. at a 1 mg/kg dose. A2aR is thought to modulate multiple inflammatory cells, and to be one of CBD’s target receptors. Cognitive and motor functions, assessed 3 weeks after the beginning of ethanol intoxication, were markedly impaired in BDL mice. CBD significantly improved these BDL-induced impairments by down-regulating TNF-α 1 receptor mRNA expression (up-regulated in BDL mice), and restoring BDNF mRNA expression (down-regulated in BDL mice). Interestingly, the effect of CBD on TNF-α receptor 1 mRNA expression was blocked by ZM241385, suggesting a CBD reduction of cerebral inflammation by regulation of the adenosine system, while it had no effect on BDNF mRNA expression.

Finally, in a hepatic encephalopathy model (Avraham et al., 2011), a single dose of thiocetamide (TAA) was administered i.p. (200 mg/kg) to mice, to induce a fulminant hepatic failure (FHF), while vehicle was injected in the control group. A single dose of either CBD (5 mg/kg) or vehicle was injected 1 day after TAA. Neurological and motor functions were assessed on day 2 and day 3, respectively. A first group of mice was sacrificed on day 4, their brain and liver were removed for histopathological analyses, and plasma liver enzymes levels were measured. Cognitive functions were tested in a second group of mice 8 days after liver failure induction, and brain 5-hydroxytryptamine (5-HT) levels were measured 12 days after the beginning of the experiment. In TAA-mice, CBD restored neurological and cognitive functions impaired by the FHF model, and partially restored motor functions. CBD restored ammonia, bilirubin and liver enzyme levels, increased by FHF, as well as 5-HT levels in the brain (increased by FHF).

In conclusion, CBD significantly reduces alcohol-induced neuronal loss after binge and chronic ethanol exposure in preclinical studies, possibly through immunomodulatory properties involving regulation of the cerebral adenosine system, and antioxidant properties. Effects of CBD on ethanol-induced clinical impairments were also associated with significant improvement in cognitive functions.

## Discussion

**Figure 1:**
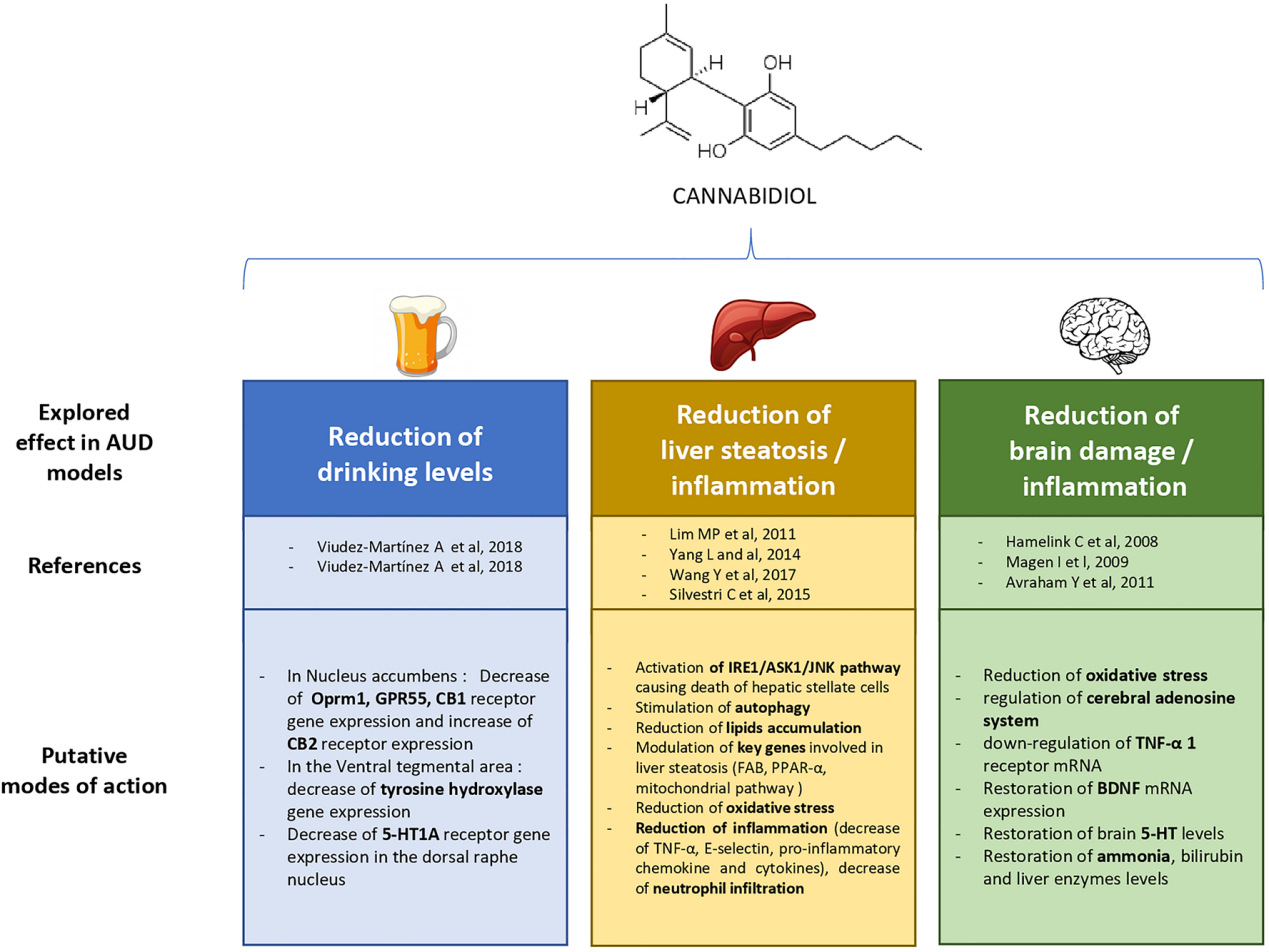
Explored therapeutic prospects of cannabidiol in previous experimental research on alcohol use disorder (AUD).

The aim of this review was to highlight, based on preclinical literature, the promising therapeutic applications of CBD in the reduction of drinking in AUD, and for improving or preventing alcohol-related damage on the liver and the brain. The main findings on these different topics are displayed in **Figure 1**. First, CBD was able to reduce motivation for alcohol, relapse, and the global level of alcohol intake in mice. Next, CBD reduced alcohol-induced liver damage, by reducing liver fibrosis *via* its immunomodulatory and antioxidant properties, as well as its action on activated HSC, stimulation of autophagy, and *via* regulation of lipid accumulation in the liver. Last, CBD acts as a multimodal neuroprotective agent that could decrease alcohol-induced neuronal damage leading to cognitive and motor impairment in animals. This latter effect could be associated with CBD antioxidant properties and immunomodulatory action, possibly correlated with the cerebral adenosine system.

Although fewer studies are available to assess the effects of CBD on cannabinoid type 2 receptors (CB2), there could be another mechanism involved in its protective effects on the liver and the brain. CB2 receptors are cannabinoid receptors that are mainly expressed in the immune system (Lotersztajn et al., 2008). CBD seems to have complex interactions with CB2 receptors, acting as a negative allosteric modulator (Martínez-Pinilla et al., 2017).

In an experimental study with cultured hepatic myofibroblasts and activated HSC from human liver biopsy (Julien et al., 2005), CB2 receptors were not detected in normal human liver whereas they were highly up-regulated in cirrhotic liver. Activation of CB2 receptor led to antifibrogenic effects by growth inhibition that probably involved cyclooxygenase-2 (COX-2), and to the increase in apoptosis by regulating oxidative stress. In the same study, mice invalidated for CB2 receptor developed enhanced liver fibrosis.

Hepatoprotective properties of CB2 receptors were also shown in a mice model of carbon tetrachloride-induced acute hepatitis (Teixeira-Clerc et al., 2010). Activation of CB2 receptors reduced liver injury and accelerated liver regeneration by immunomodulation involving TNF-α, IL-6, matrix metallo-proteinase-2 (MMP-2), and reduction of oxidative stress.

In another animal model (Louvet et al., 2011) of alcohol-fed mice, CB2 receptors regulated Kupffer cells polarization by provoking a switch from a classical proinflammatory program of activation (M1) to an alternative anti-inflammatory one (M2). This eventually protected the liver from the deleterious effects of alcohol. Moreover, in the same study, CB2 receptors were shown to reduce steatosis based on paracrine effects of Kupffer cells on hepatocytes.

Finally, as far as the brain is concerned, specific pharmacological activation of CB2 receptors in a forced alcohol consumption rat model rescued alcohol-induced impaired neural progenitor cells (NPC) proliferation, thus counteracting alcohol-induced neuronal damage (Rivera et al., 2015).

However, all these promising findings come from animal models only, and there are currently no results from clinical trials studying CBD in human AUD. It should be noted, however, that one double-blind randomized clinical trial is currently being conducted in the United States. In this ongoing study, CBD is administered *versus* placebo to patients with AUD, with the aim of reducing the overall level of alcohol drinking (NCT03252756).

Having a similar effect as drugs such as nalmefene (Mann et al., 2016), baclofen (Agabio et al., 2018), or topiramate (Palpacuer et al., 2018), CBD might thus be another good candidate molecule for reducing drinking in subjects with AUD. Furthermore, the antioxidant and immunomodulatory properties of CBD constitute additional and valuable features in the achievement of harm reduction in subjects with AUD, *via* a reduction or even a prevention of alcohol-related liver or brain damage. While specific pharmacological strategies of harm reduction have previously been developed in other substance use disorders, in particular in opioid use disorder, no other drug has been used in AUD for the specific purpose of reducing alcohol-related damage, even without drinking reduction.

Moreover, CBD seems to have other interesting harm reduction properties, which have not been assessed in AUD models so far, and thus, could not be investigated in this review. CBD has well-known antiepileptic properties: in 2018, the Food Drug Administration (FDA) granted an approval to CBD (EPIDIOLEX^®^, GW Pharmaceuticals), for Dravet and Lennox–Gastaut syndromes. Given that patients with AUD are at increased risk to exhibit alcohol-induced or withdrawal-related seizures, CBD could prevent the occurrence or reduce the severity of seizures in this population. CBD also possesses anxiolytic and analgesic properties (Carrier et al., 2006; Rock et al., 2012; Mori et al., 2017). Since subjects with AUD display anxious symptoms or chronic pain more frequently than subjects without AUD (Schuckit and Hesselbrock, 1994; Witkiewitz and Vowles, 2018), CBD could reduce the overall level of anxiety and pain in subjects with AUD, which could improve overall outcomes such as stress and quality of life. Indeed, 5-HT receptors, which are known to regulate anxiety (Ishikawa and Shiga, 2017; Batista and Moreira, 2019; Wang et al., 2019), are one of CBD’s targets (Russo et al., 2005; Pazos et al., 2013), and were studied in AUD (Underwood et al., 2018). For example, ondansetron, a 5-HT3 receptor antagonist, showed some efficacy in both preclinical and clinical studies on AUD (Johnson et al., 2013; Moore et al., 2014; Soyka and Müller, 2017). More recently, a preclinical study in mice (Viudez-Martínez et al., 2018) showed that the 5-HT1a receptor antagonist WAY100635, blocked the positive effect of a CBD-plus-naltrexone combination on motivation and ethanol intake.

Anxiolytic properties of CBD could also be explained by its potential ability to regulate endocannabinoid levels. FAAH is an enzyme responsible for the degradation of endocannabinoids such as anandamide and 2-arachidonoylglycerol (Watanabe et al., 1998; Bisogno et al., 2002; Leweke et al., 2012; Deutsch, 2016), after they have bound to fatty acid binding protein (FABP). CBD inhibits FAAH and thus, prevents anandamide from being degraded (de Filippis et al., 2008; De Petrocellis et al., 2011; Leweke et al., 2012; Stern et al., 2017). Facilitation of endocannabinoid signaling by repeated administration of CBD led to a decrease of chronic stress in mice (Campos et al., 2013). In a human study with a simulated public speaking test in patients suffering from social phobia, CBD was found to significantly reduce anxiety (Bergamaschi et al., 2011). Contrasting with these results, a preclinical study in rats showed an impaired FAAH function in the alcohol-preferring phenotype compared to the nonpreferring phenotype, causing an overreactive endocannabinoid transmission and a compensatory down-regulation of CB1 signaling (Hansson et al., 2007). However, extrapolating all these results in humans seems quite premature: for example, an experimental study on human cells found that CBD had no action on FAAH but rather targeted several types of FABPs (Elmes et al., 2015).

Finally, in addition to hepatic and brain damage, alcohol induces many other noxious effects on the body, for example by inducing alcohol-related myocarditis, or various types of cancers. Because of its immunomodulatory properties, protective effects of CBD against these other harms should be further investigated in both animals and humans. Overall, CBD safety aspects appear to be good, which is another important criterion for extending human research to patients with AUD. So far, no severe clinical states resulting from CBD intoxication have been reported, neither in animal nor in human use. Similarly, to our knowledge, no pharmacological tolerance, withdrawal syndrome, abuse, or addictive behaviors, have been reported hitherto. This is an important factor to consider before using CBD in AUD or other addictive disorders.

Despite the multiple prospects of CBD in AUD that have been emphasized in this review, many issues and unsolved questions remain. The current literature only pertains to animal models, and the translational aspects of the findings listed in this review are yet to be established. Moreover, CBD effective dose range observed in animals is unlikely to be similar in humans. This point is important because the dose–effect relationship of CBD depends on the type of effect and is not always linear. For example, some effects of CBD seem to have an inverted U-shaped dose–response curve. Regarding anxiety, while a dose superior to 20 mg/kg appears to be ineffective in animals (Guimarhes et al., 1990), a human study with a Simulating Public Speaking Test confirmed this U-shaped dose–response, with an efficacy observed with 300 mg of CBD, but not with 150 mg or 900 mg (Linares et al., 2016). However, other animal studies found an anxiolytic effect with repetitive doses of 30 mg/kg, which may activate different pathways (Campos et al., 2013; Fogaça et al., 2018). In animal models of depression, a dose of 30 mg/kg of CBD was found to be as effective as tricyclic antidepressants whereas a 100 mg/kg one was ineffective (Zanelati et al., 2010). With higher doses, activation of TRPV1 reduced the anxiolytic/antidepressant effect (Campos and Guimarães, 2009). Higher doses of CBD (800 mg/kg, 1,000 mg/day) seem to be needed to obtain antipsychotic effect with reduction of positive psychotic score in clinical studies (Leweke et al., 2012; McGuire et al., 2018). Consistent with its large therapeutic target spectrum, sometimes with opposite effects, the therapeutic dose range of CBD should be defined specifically for the various symptoms that clinicians want to alleviate, in connection with hypothetical receptors or secondary pathways.

In conclusion, experimental data underline that CBD offers multiple therapeutic prospects in patients with AUD. CBD seems to facilitate drinking reduction, making CBD an interesting pharmacological option in AUD treatment. Moreover, CBD might provide idiosyncratic protection to the liver and the brain, which could reduce the development and impact of both ARLD and ARBI. In this perspective, CBD treatment could be proposed to subjects who are unable to reduce or to stop alcohol consumption, in order to prevent or reduce the effects of alcohol on the brain and the liver, thus opening new and original therapeutic options for harm reduction in AUD. CBD could have many more positive effects in subjects with AUD, including antiepileptic, cardioprotective, anxiolytic, or analgesic ones. Human studies are thus crucially needed to explore the many prospects of CBD in AUD and related conditions.

## Author Contributions

BR conceived the presented idea and supervised the project. MNa, MNo, BR, and JD wrote the manuscript. All authors provided critical feedback and helped shape the manuscript. All authors approved the final version for submission.

## Funding

Funds for open access publication fees were provided by CH le Vinatier.

## Conflict of Interest Statement

The authors declare that the research was conducted in the absence of any commercial or financial relationships that could be construed as a potential conflict of interest.
